# Targeting an essential viral oncoprotein with an IL-7-enhanced mRNA vaccine induces durable immunity to Merkel cell carcinoma

**DOI:** 10.1016/j.celrep.2025.116359

**Published:** 2025-10-01

**Authors:** Alexander Frey, Kathryn Clulo, Yuewei Fei, Therese Cordero Dumit, Frankie Scallo, Jerry William Allen, Emily Chang, Curtis J. Perry, Lena V. Wirth, Daniel Jacobs, David A. Braun, Marcus W. Bosenberg, Thuy T. Tran, James Clune, Harriet M. Kluger, Kelly Olino, Jeffrey J. Ishizuka

**Affiliations:** 1 Department of Surgery, Yale School of Medicine, New Haven, CT 06511, USA; 2 Department of Medicine (Oncology), Yale School of Medicine, New Haven, CT 06511, USA; 3 Department of Immunobiology, Yale School of Medicine, New Haven, CT 06511, USA; 4 Department of Pathology, Yale School of Medicine, New Haven, CT 06511, USA; 5 Department of Dermatology, Yale School of Medicine, New Haven, CT 06511, USA; 6 Present address: School of Medicine, Uniformed Services University; Bethesda, MD, 20814, USA; 7 Lead contact

## Abstract

Although mRNA technologies have reinvigorated cancer vaccine development, the identification of strong antigens with consistent tumor cell expression and generation of durable antigen-specific CD8^+^ T cell memory remain key challenges. We identified the Merkel cell carcinoma (MCC) large T antigen (LTA) as an optimal vaccine target, essential for tumor cell survival and immunogenic in a cancer with high unmet clinical need. We developed an mRNA vaccine to MCC-LTA in murine studies and patient samples. We showed that antigen loss develops rapidly and causes resistance in mouse models when immunogenic, but non-essential antigens are targeted. To improve T cell response durability, we co-encoded LTA and IL-7, co-localizing proliferative and memory signals spatially and temporally with antigen exposure. IL-7-containing mRNA vaccines improved antigen-specific T cell expansion, memory differentiation, and tumor control. We propose that the principles of antigen essentiality and memory signal co-encoding may be adapted to improve the efficacy of mRNA therapeutics.

## In brief

Frey et al. develop an mRNA immunotherapy for Merkel cell carcinoma, a lethal neuroendocrine skin cancer with high unmet medical need. They target an essential viral oncoprotein with an IL-7-enhanced vaccine, overcoming two central challenges in the development of therapeutic cancer vaccines: antigen loss and CD8^+^ T cell response durability.

## Graphical Abstract

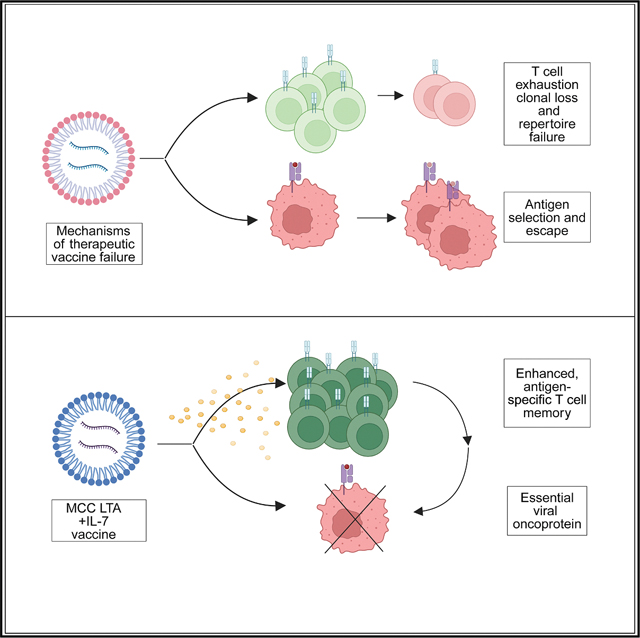

## INTRODUCTION

The clinical generation of mRNA vaccines during the global SARS-CoV-2 pandemic accelerated the ongoing development of these technologies as therapeutic cancer vaccines.^1–3^ However, in contrast to vaccines against pathogens, cancer vaccine therapies face unique challenges, including increased reliance on CD8^+^ T cells as opposed to antibody-mediated immunity and long-term sustainment of T cell response.^4,5^ Further, unlike coronavirus vaccines, the optimal target antigen for cancer vaccines can vary by tumor type and individual tumor, with a recent focus on patient-specific tumor mutation-associated neoantigens. Although algorithms for predicting neoantigens have rapidly improved, most neoantigens are not immunogenic.^6,7^ Further, neoantigens are frequently heterogeneously expressed within primary patient tumors and at metastatic sites.^8^ In contrast to neoantigen-directed vaccines, vaccines for virus-associated cancers have the advantage of targeting intrinsically immunogenic proteins that are not subject to central tolerance. Moreover, because viruses are frequently involved in early and genetically bottlenecked stages of oncogenesis, the expression of viral target antigens is frequently homogeneous.

Merkel cell carcinoma (MCC) is a rare, aggressive neuroendocrine cancer that is, stage for stage, more lethal than any other skin cancer.^9^ Although its cell of origin is unknown, MCC is caused by either ultraviolet radiation, or, in 80% of US cases, by Merkel cell polyomavirus (MCPyV). Although immune checkpoint inhibitors (ICIs) have improved the prognosis of MCC, only ~50% of patients have responses to treatment, and responses are frequently transient.^10^ Other systemic therapies, including chemotherapy, have only limited benefit.^11^ Virus-associated MCC is associated with genomic integration of a truncated form of the viral T antigens (TAs), including the large T antigen (LTA) and its isoform, the small T antigen. In contrast to most tumor antigens, continued expression of the TA proteins and LTA specifically are essential for tumor cell viability.^12–14^ Further, several studies have identified LTA-specific T cells in the blood of patients with virus-associated MCC.^15–17^ Given its differentiated properties, we hypothesized that the MCC LTA may serve as an optimal target for mRNA vaccination. Here, we develop an LTA-targeting mRNA vaccine in murine models and MCC patient samples. We demonstrate effective treatment, including the accumulation of dendritic cells in vaccine-draining lymph nodes and activated T cells within the tumor microenvironment in mouse models. We model the effects of antigen loss on vaccine efficacy and show rapid outgrowth under conditions in which the antigen is non-essential for tumor cell survival. In patient samples, *ex vivo* vaccination induced cytotoxic and proliferative transcriptional states, as well as the enrichment of LTA-specific T cell clonotypes. Finally, to address the challenge of sustaining T cell responses, we developed vaccines that co-encode the cytokine signal IL-7 and demonstrate improved memory formation and tumor control. In summary, we advance a therapeutic option for a lethal cancer with high unmet need in murine models and patient samples and develop approaches that may be used broadly to improve the therapeutic efficacy of mRNA cancer vaccines.

## RESULTS

### An mRNA vaccine targeting the MCC-LTA enhances tumor immunity and PD-1 response

Although MCC is a rare cancer, its incidence and prevalence have increased significantly over the past 20 years. To assess the current and emerging burden of MCC, we revisited a cross-sectional retrospective analysis of the Surveillance, Epidemiology and End Results Program database, including 11,340 patients with MCC. In line with prior predictions, the current incidence is estimated to have increased from 0.460 (95% CI: 0.413–0.517) to 0.673 (95% CI:0.623–0.725) per 100,000 between 2000 and 2021 (Figure S1A), with a sharp increase in reported cases that leveled off during the COVID-19 pandemic, consistent with many other cancer diagnoses. This increase underscores the need for new treatment and prevention approaches.

Given the rising unmet medical need in virus-associated MCC and advances in mRNA vaccine technologies, we sought to test whether an mRNA vaccine approach could be used therapeutically to treat and prevent the recurrence of this disease or prophylactically in subsets of patients at high risk for developing MCC. We selected the MCC-LTA as the target of our vaccine given its established immunogenicity in patients, its universal expression in virus-associated MCC, and evidence that loss of its expression is lethal to MCC tumor cells *in vitro* and *in vivo.*^12–14^ To identify the optimal LTA sequence for vaccination, we selected the consensus overlap LTA sequence from published patient cohorts.^18–20^ We combined this sequence with the other necessary components of functional mRNA (Figure S1B), including a synthetic 5′ UTR sequence identified in a translation efficiency screen performed by Cao et al. and the hemoglobin subunit alpha 1 3′ UTR, which was selected due to its short length and success in prior mRNA functional constructs.^21–23^ We hypothesized that translation efficiency and protein trafficking could be enhanced by inclusion of a signal peptide (SP) sequence from the SARS-CoV-2-spike protein and consequently made vaccine variants with and without the SP for further evaluation.^24–27^ Specifically, we reasoned that SP inclusion would preferentially localize the LTA oncoprotein within the cytoplasm and secreted fraction, limiting nuclear access and opportunities for interaction with the RB1 protein. Furthermore, we reasoned that SP inclusion would enhance secretion and downstream cross-presentation of the LTA by classical type I dendritic cells (cDC1s). Following *in vitro* transcription, we transfected HEK293T cells with vaccine variants with SP-containing and SP-null vaccines. In the setting of Golgi block, we observed comparable expression of the LTA protein in SP- and non-SP-containing variants by western blot (Figure S1C). However, consistent with our expectation, SP-containing variants localized to the cytoplasm, whereas non-SP variants were both nuclear and cytoplasmic (Figure S1D).

**Figure 1.**
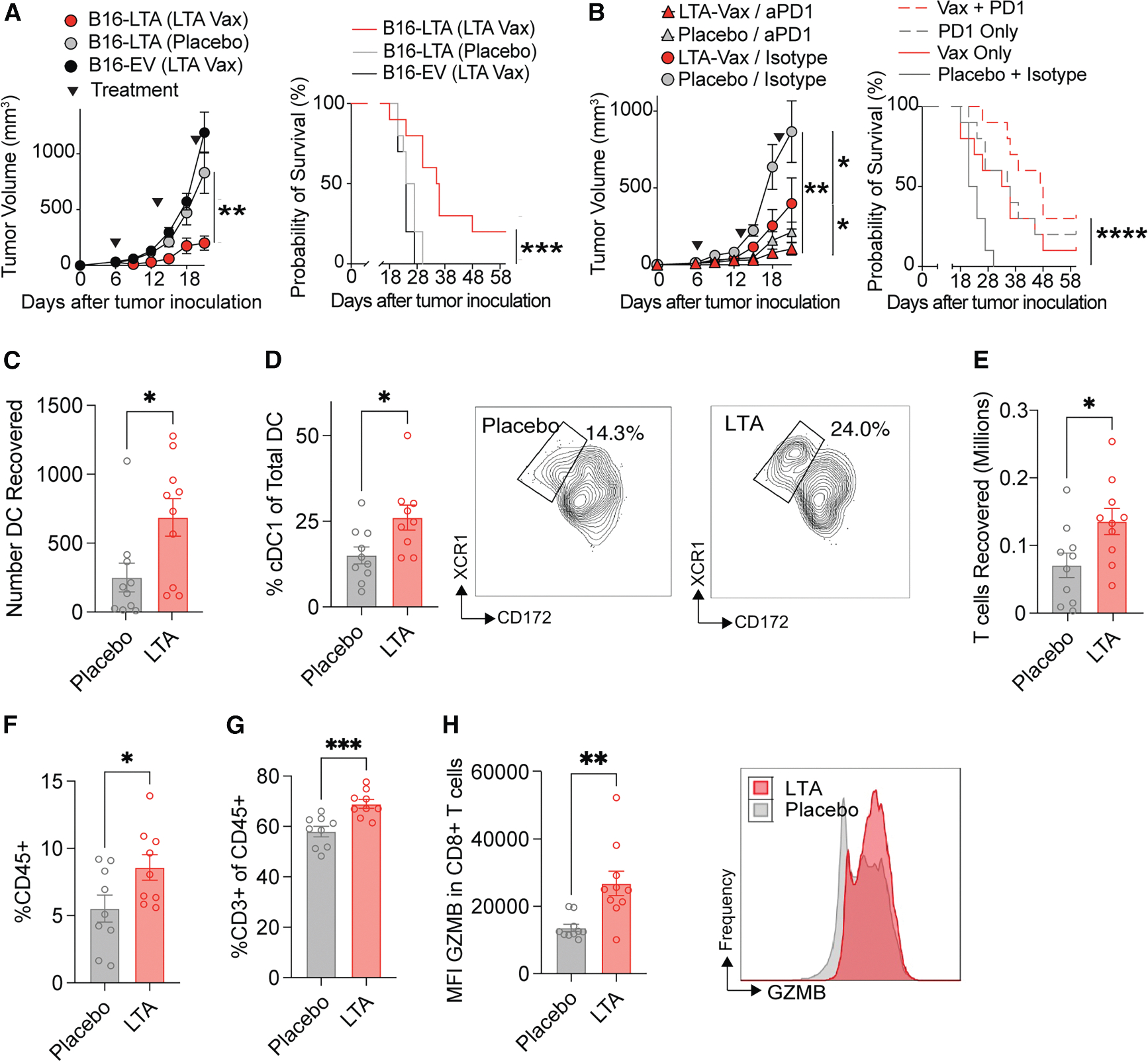
LTA-targeting mRNA vaccine improves anti-tumor immunity in an aPD1-resistant mouse model (A) Tumor growth curves comparing treatment with 15 μg LTA mRNA vaccine (red) or placebo LNP (gray) injected into the gastrocnemius muscle contralateral to tumor implantation in the pLX311-LTA model as well as treatment in the pLX311-EV model. Treatment was given on d6, 13, and 20; *N* = 10 per group (left); statistical comparison by Student’s t test. Survival curves for mice demonstrating increased median survival with 15 μg LTA mRNA treatment in the pLX311-LTA model. *N* = 10 per group (right); statistical comparison by Mantel-Cox test. All mice were followed a minimum of 60 d, at which point, surviving mice were free of detectable tumors. (B) Tumor growth curves comparing single-agent LTA mRNA treatment (red circles), single-agent anti-PD1 treatment (gray triangles), combination LTA mRNA + anti-PD1 (red triangles), and placebo vaccine + isotype antibody (gray circles) in the pLX311-LTA model (left); statistical comparison by Student’s t test. Associated survival curves following LTA vaccine or aPD1 treatment (right); statistical comparison by Mantel-Cox test. (C) Absolute number of dendritic cells recovered from vaccine-draining lymph nodes from placebo (gray) and LTA vaccine-treated (red) mice measured by flow cytometry. (D) Percentage of classical type I dendritic cells (cDC1s) and gating strategy in placebo- and LTA vaccine-treated mice. (E) Total T cells recovered from vaccine-draining lymph nodes in placebo and LTA vaccine-treated mice. (F) Proportion of CD45^+^ immune cells recovered from tumors following placebo LNP and LTA vaccine treatment. (G) Proportion of CD3^+^ T cells as a percentage of CD45^+^ immune cells from tumors following placebo LNP and LTA vaccine treatment. (H) Mean fluorescent intensity of granzyme B within CD8^+^ T cells from tumors following placebo LNP and LTA vaccine treatment. For tumor growth and survival experiments, 1 million tumor cells were injected subcutaneously and vaccine or placebo treatment was given on d6, 13, and 20, while anti-PD1 treatment or isotype antibody was given by intraperitoneal injection on d6, 9, and 12. *N* = 10 per group. For flow cytometry experiments, mice were challenged with LTA-expressing tumors on d0 and treated with placebo LNP (gray) or LTA mRNA vaccine (red) on d6 and 13. Tumors, vaccine-, and tumor-draining lymph nodes were collected on d15. *N* = 10 per group. Data represented in growth curves and bar plots are mean ± SEM; statistical comparison in all bar plots are by Student’s t test. *p value < 0.05 and ≥ 0.01, **p value < 0.01 and ≥ 0.001, ***p value < 0.001 and ≥ 0.0001, and ****p value < 0.0001.

To enable *in vivo* evaluation of our vaccine candidate, we enforced the expression of the MCC-LTA in B16 melanoma cells using lentiviral vectors (B16-LTA) and confirmed expression by western blot compared with empty vector controls (B16-EV; Figure S1E). Because the expression of an immunogenic, foreign protein has the potential to alter tumor growth kinetics and viability, we compared the growth of B16-LTA, B16-EV, and parental B16 tumors and found them to be equivalent (Figure S1F). To test the efficacy of the LTA mRNA vaccine, we treated B16-LTA tumors with 15 μg of mRNA vaccine encapsulated in SM-102-based lipid nanoparticles (LNPs) or volume-matched LNP placebo by intramuscular injection to the contralateral gastrocnemius on d6, 13, and 20 after confirming tumor palpability prior to initiating treatment. LTA vaccine treatment resulted in significant tumor growth suppression and improved survival in B16-LTA tumors compared with placebo controls, with all surviving mice 60d following treatment cleared of tumors (Figure 1A). Notably, the vaccine had no effect in B16-EV tumors that lack LTA expression, suggesting a lack of effect from stimulating innate immunity with mRNA and LNPs alone. To confirm the effect of vaccination, we tested a single-dose treatment on d6 and d9 after tumor implantation, observing comparable results in each setting (Figure S1G). Given that aPD-1 is the backbone of systemic therapy in MCC and has shown promise in combination with mRNA vaccines, we next tested whether our vaccine could sensitize tumors to PD-1 ICI. Indeed, while both aPD-1 and LTA vaccine were effective against B16-LTA tumors, the combination of both treatments improved tumor control and survival compared with either treatment alone (Figure 1B).

### mRNA vaccination reprograms tumor and lymph node microenvironments

To test the mechanism by which the LTA mRNA vaccine suppressed tumor growth and prolonged survival in LTA-tumor-bearing mice, we performed *ex vivo* analysis of tumor and lymph node tissues using flow cytometry. On d15 after tumor implantation, following two doses of LTA vaccine or placebo LNP, we sacrificed mice and dissected tumors, tumor-draining, and vaccine-draining lymph nodes. LTA vaccine-draining lymph nodes were larger and contained a greater number of cells (Figure S2A), including a greater abundance of dendritic cells (DCs; Figures 1C and S2B) and a higher proportion of classical cDC1s (Figure 1D) than did controls. Notably, LTA vaccine-draining lymph nodes contained a greater total abundance but a smaller proportion of CD3^+^ T cells and granzyme B (GZMB) expression in CD8^+^ T cells (Figures 1E, S2C, and S2D), reflecting the proliferative and trafficking dynamics of these populations. Consistent with tumor size results, the mass of tumors from vaccine-bearing mice was significantly smaller than the mass of d15 tumors from control mice (Figure S2E). Within tumors, we observed an increase in overall immune infiltration (Figure 1F), CD3^+^ T cells (Figures 1G and S2F), and GZMB expression in CD8^+^ T cells (Figure 1H). In contrast to vaccine-draining lymph nodes, we did not observe any significant difference in the immune composition of LTA vaccine-compared with placebo LNP-treated tumor-draining lymph nodes (Figures S2C, S2D, and S2G). Overall, vaccination remodeled the immune microenvironment in the tumor and draining lymph node to support the expansion of CD8^+^ T cell populations.

### Antigen loss underlies therapeutic resistance to vaccination

**Figure 2.**
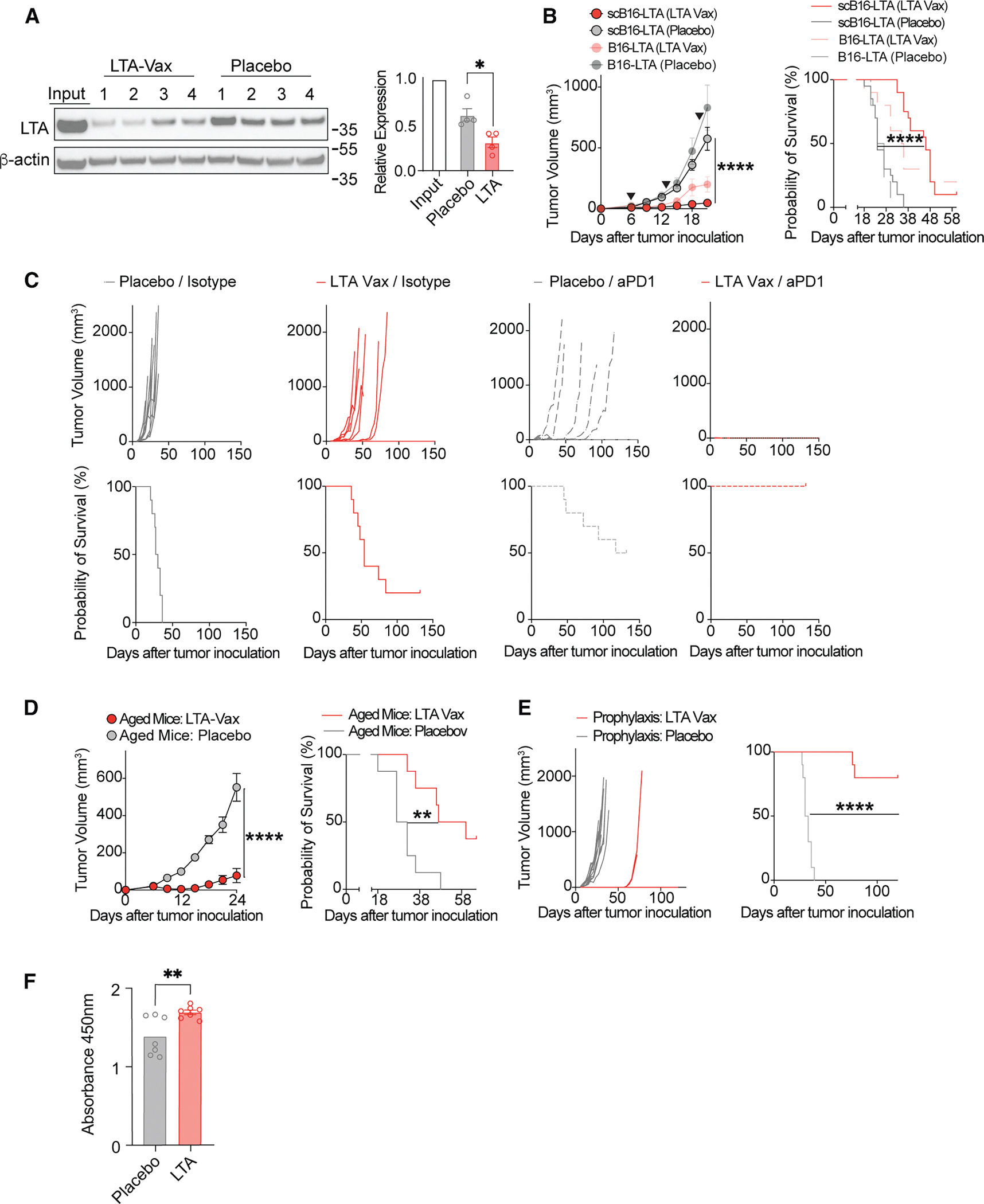
Loss of antigen underlies vaccine resistance in murine models (A) At the time of tumor survival endpoint, mice were sacrificed, and tumors were re-isolated and lysed for the measurement of protein expression. Western blot of LTA expression in input B16-LTA cells and re-isolated tumors from LTA vaccine- and placebo LNP-treated mice are depicted along with quantification using ImageJ. (B) LTA vaccine (red) and placebo LNP (gray) treatment models were repeated as per Figure 1B, comparing bulk (lighter colors) with a single-cell-cloned B16-LTA model and using tumor growth and survival endpoints. Growth curve statistical comparison by Student’s t test, and survival by Mantel-Cox test. (C) Single-cloned B16-LTA tumors were treated with either LTA vaccine (red) or placebo LNP (gray) on d6, 13, and 20 and with either aPD1 antibody (dashed lines) or isotype control (solid lines) on d6, 9, and 12. Tumor growth curves are depicted. (D) Mice were aged 68 weeks, and tumor challenges were repeated as in (B). Growth curve statistical comparison by Student’s t test, and survival by Mantel-Cox test. (E) Mice were treated with prophylactic vaccination regimen, including 15 μg d-60, d-54, and d-30 prior to tumor challenge with 1 million cells subcutaneously. Individual tumor growth curves for LTA vaccine treatment (red) and placebo LNP treatment (gray) are depicted, as well as survival curves for mice. Statistical comparison for survival curves by Mantel-Cox test. (F) LTA ELISA was constructed by immobilizing the LTA protein on the surface of a cell culture plate. Serum from mice that had undertaken prophylaxis regimen using LTA vaccine (red) or placebo LNP (gray) in (E) is depicted. Data represented in growth curves and bar plots are mean ± SEM; statistical comparison for all bar plots is by Student’s t test. *p value < 0.05 and ≥ 0.01, **p value < 0.01 and ≥ 0.001, ***p value < 0.001 and ≥ 0.0001, and ****p value < 0.0001.

The MCC-LTA was selected as an example of a class of vaccine targets that are essential for tumor cell survival. Given that B16 tumor growth is not dependent on LTA expression (Figure S1E), unlike human MCC, we hypothesized that antigen loss may limit vaccine efficacy in a population of tumor cells with heterogeneous target antigen expression. To test this hypothesis, we repeated the vaccination of LTA-tumor-bearing mice and re-isolated tumor cells at the time of tumor outgrowth. Indeed, tumors that grew out following LTA vaccine treatment had significantly lower expression of the LTA than did placebo-LNP-treated tumors, and both had lower expression of the LTA than the input tumor cell line (Figure 2A). To limit antigen loss, we performed single-cell cloning by limiting the dilution of LTA-expressing tumor cells and selected a tumor cell line with uniform, high expression of the target antigen (Figure S2H). This model was then used to test LTA mRNA efficacy as a monotherapy and in combination with aPD-1. Compared with prior treatment results, LTA mRNA control of tumor growth and survival endpoints were improved in the LTA-high, single-cloned model compared with the bulk LTA-expressing population (Figure 2B). Moreover, using this model, we observed that LTA vaccination enhanced sensitization to aPD-1 treatment and resulted in uniform cure of tumors (Figure 2C).

Given that MCC is prevalent in elderly patients and that immunosenescence has the potential to compromise anti-tumor T cell responses,^28,29^ we tested whether our mRNA vaccine would be effective in mice aged 68 weeks. Notably, the vaccine performed equally well in aged compared with young mice against our single-cloned model (Figure 2D), suggesting the potential for translation in aged patient populations.

In addition to its therapeutic effects, we hypothesized that LTA vaccination may confer prophylactic immunity, relevant to populations at high risk of MCC development, such as chronic lymphocytic leukemia patients, and could be used for secondary prevention in the adjuvant setting for patients with treated MCC at risk for recurrence. To test this hypothesis, we treated non-tumor-bearing mice with three doses of LTA vaccine (d-60, d-54, and d-30 prior to challenge). 30 days following the final dose, we challenged mice with one million tumor cells (Figure 2E). Prophylactic vaccination with LTA mRNA resulted in rejection of 80% of tumors, compared with 0% with placebo control, supporting the generation of antigen-specific memory. In the minority of cases in which tumors formed following LTA vaccination, their growth was controlled for more than 60 days after tumor cell injection, at which time, all placebo mice had reached endpoint (Figure 2E). In addition to tumor rejection, we observed the development of LTA-specific antibodies in vaccinated compared with control mice (Figure 2F).

### *Ex vivo* vaccination enhances tumor-specific T cell responses in MCC patient samples

**Figure 3.**
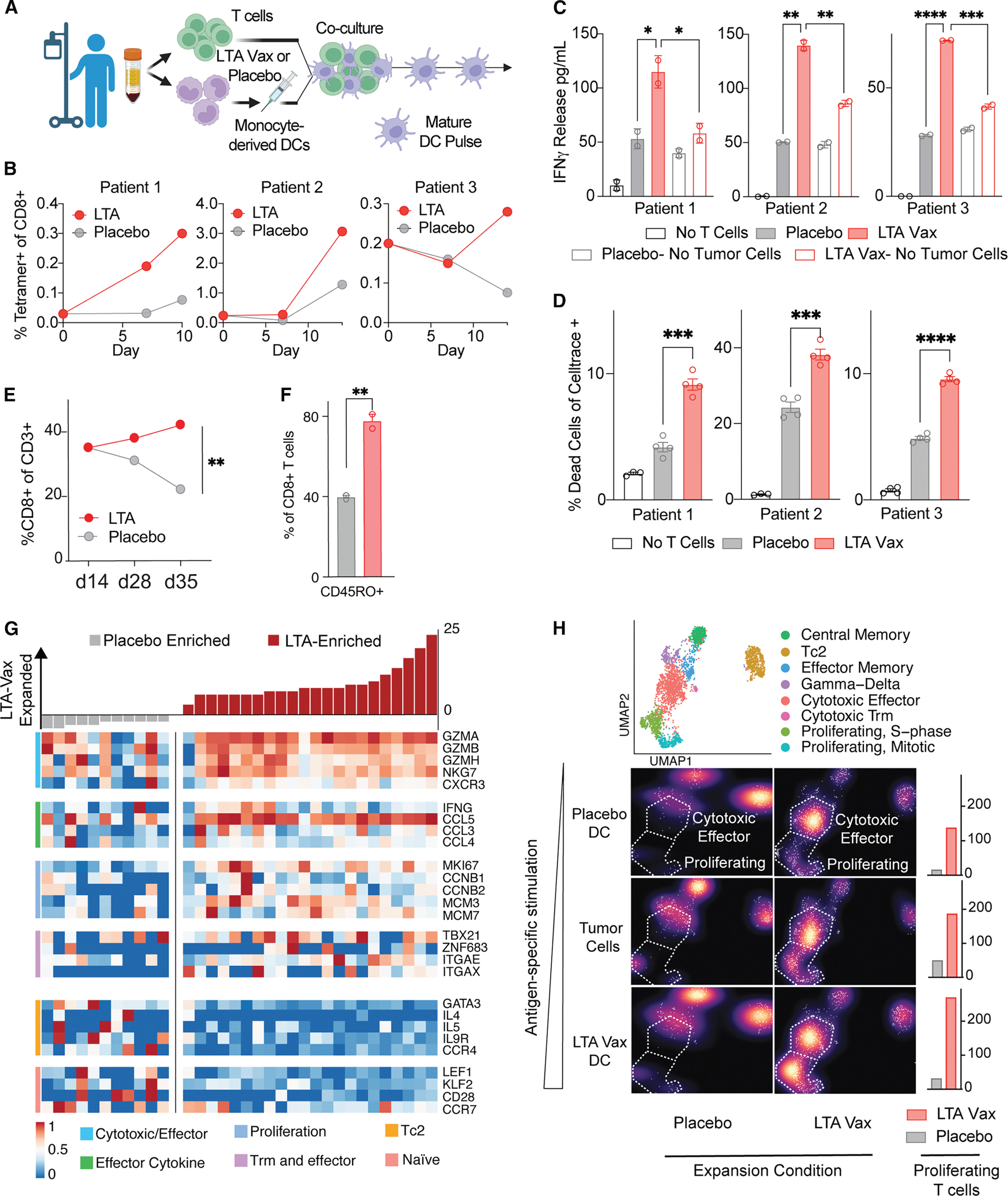
*Ex vivo* LTA vaccination improves MCC patient LTA and tumor-specific T cell responses (A) Schema for *ex vivo* vaccination. MCC patient PBMCs are isolated, and T cell- and monocyte-derived dendritic cell (moDC) cultures are separately maintained. moDCs are transfected with LTA vaccine or placebo and used weekly to stimulate T cell cultures for up to 30 days. (B) Expansion of antigen-specific CD8^+^ T cells identified by the HLA-A2 tetramer for LTA epitope SMFDEVDEA in PBMCs co-cultured with LTA antigen-loaded moDCs (red) vs. placebo (gray). Tetramer expansion is depicted for three separate HLA-02*01 MCPyV+ MCC patients. Patients are matched for B, C, and D. (C) ELISA results for the detection of IFNγ following 24 h co-culture of LTA vaccine (red, solid bars) or placebo (gray, solid bars) PBMCs with HLA-matched MCC tumor cells. T cells were expanded for 14 days following the procedure in (A). LTA and placebo-expanded T cells cultured without tumor cell stimulation (red, empty bars; gray empty bars) are depicted as controls. (D) Mean percent cell death in HLA-A*02:01-matched MCC tumor cells by flow cytometry following 24 h of co-culture with LTA vaccine (red) or placebo (gray) PBMC pools on d14 of expansion using a 5:1 effector-to-target ratio. (E) Proportional expansion of CD8^+^ T cells in culture for patient 4 following stimulation with LTA vaccine (red) compared with placebo (gray). Statistical comparison is by Student’s t test. (F) Expression of the memory marker CD45RO on d28 of the expansion protocol with LTA vaccine (red) or placebo (gray) moDCs. (G) Enrichment of CD8^+^ T cell clonotypes following LTA compared with placebo moDC stimulation. Enrichment is calculated as frequency in LTA minus frequency in placebo conditions. Mean gene expression by clonotype is shown for selected differential genes following overnight stimulation of LTA and placebo cultures with patient-matched tumor cells. (H) Distinct cytotoxic and proliferative CD8^+^ T cell states detected following vaccine or placebo LNP expansion (left). Enrichment of proliferative states following the stimulation of placebo-expanded (left) or LTA-expanded (right) cultures for 24 h with either placebo-loaded DCs (top), matched tumor cells (middle), or LTA-loaded DCs (bottom). Data represented in bar plots are mean ± SEM; statistical comparison for all bar plots is by Student’s t test. *p value < 0.05 and ≥ 0.01, **p value < 0.01 and ≥ 0.001, ***p value < 0.001 and ≥ 0.0001, and ****p value < 0.0001.

Given that our murine studies were conducted in a modified melanoma model, we sought to validate our vaccine directly in samples from MCC patients. To assess the potential for clinical translation of the LTA mRNA vaccine, we performed *ex vivo* vaccination of PBMCs from MCC patient peripheral blood (Figure 3A; Table 1). To expand and activate MCPyV^+^ MCC patient LTA-specific T cells, we pulsed patient PBMCs with either LTA mRNA or placebo-transfected, matched, activated monocyte-derived DCs (moDCs) weekly for up to 35 days. Prior to pulses, we confirmed moDC differentiation and cytokine-mediated activation by flow cytometry and LTA expression by western blot (Figures S3A and S3B). Using LTA-specific MHC class I tetramers for the HLA-A*02:01 epitope SMFDEVDEA, we confirmed an increase in the proportion of antigen-specific CD8^+^ T cells in three separate patients following stimulation with LTA mRNA compared with placebo moDCs (Figures 3B and S3C). To determine the effect of LTA mRNA on T cell function, we collected supernatants from LTA or placebo-loaded moDC-PBMC co-cultures derived from the same three patients 3 days following each pulse and measured IFNγ via ELISA as a marker of favorable moDC-T cell interactions. LTA mRNA-loaded moDC co-cultures released consistently more IFNγ than controls (Figure S3D). After 14 days of moDC-stimulation, we co-cultured LTA mRNA and placebo patient PBMCs at 5:1 effector-to-target ratio with HLA-matched MCC tumor cells for 24 h. Supernatant collected from this experiment revealed that IFNγ release was increased with LTA vaccination compared to placebo control and that increased release was specific to tumor-stimulated conditions (Figure 3C). In a matched experiment, a flow cytometry-based killing assay showed increased tumor cell killing in the LTA compared with placebo co-culture (Figure 3D). These results suggest that our mRNA vaccine expanded, activated, and enhanced the functional and anti-tumor properties of patient-derived peripheral blood T cells.

For one patient, we were able to collect matched PBMCs and primary tumor cells isolated from a clinically indicated surgical resection under an IRB-approved protocol. In this case, we observed a striking outgrowth of CD8^+^ compared with CD4^+^ T cells upon repeated culture stimulation with LTA mRNA compared with placebo moDCs. This was accompanied by enrichment of activated and memory subsets by flow cytometry (Figures 3E and 3F). On d14 of our T cell expansion protocol, we restimulated this patient’s T cell cultures with matched tumor cells and observed increased IFNγ release and tumor cell killing in LTA compared with placebo-expanded cultures (Figures S3E). To determine the transcriptional states and associated TCR clonotypes of T cells expanded by the LTA mRNA vaccine, we performed matched TCR and single cell RNA sequencing on d25 of *ex vivo* vaccination. 24 h prior to submitting for sequencing, we restimulated a subset of LTA and placebo-expanded cultures with either LTA mRNA or placebo LNP-loaded moDCs or matched tumor cells. Comparing all LTA mRNA to placebo expanded T cells, we observed the emergence of a polyclonal immunodominant T cell clonotypic response, with accompanying enrichment for signatures of cytotoxicity, proliferation, and effector function compared with placebo-expanded T cells (Figure 3G). Globally, LTA-expanded T cells were notable for an enrichment of CD8^+^ compared with CD4^+^ T cells and the induction of cytotoxic functional and proliferative states compared with controls (Figures S4A–S4F). These states included the enrichment of effector cytokine genes, including *IFNG* and *XCL1*; multiple granzyme genes, *PRF1*; and a proliferative state, including *MKI67*, among others. We observed a progressive induction of cytotoxic states when LTA-expanded T cells but not placebo controls were stimulated with antigen-specific stimuli, including patient-matched tumor cells and LTA-loaded DCs (Figure 3H). To confirm that vaccination expanded antigen-specific T cells, we compared the clonotypes preferentially expanded with published TCRs from virus-positive and virus-negative patients using GLIPH2.^30^ Consistent with our expectation, the public clonotypes we observed preferentially overlapped with clonotypes from virus-positive compared with virus-negative patients (Figure S5A). These data thus support the potential for T cell expansion and the priming for activated transcriptional states, including cytokine secretion and tumor cell killing following LTA mRNA vaccination.

### IL-7-encoded mRNA vaccines improve T cell expansion, memory, and anti-tumor response

**Figure 4.**
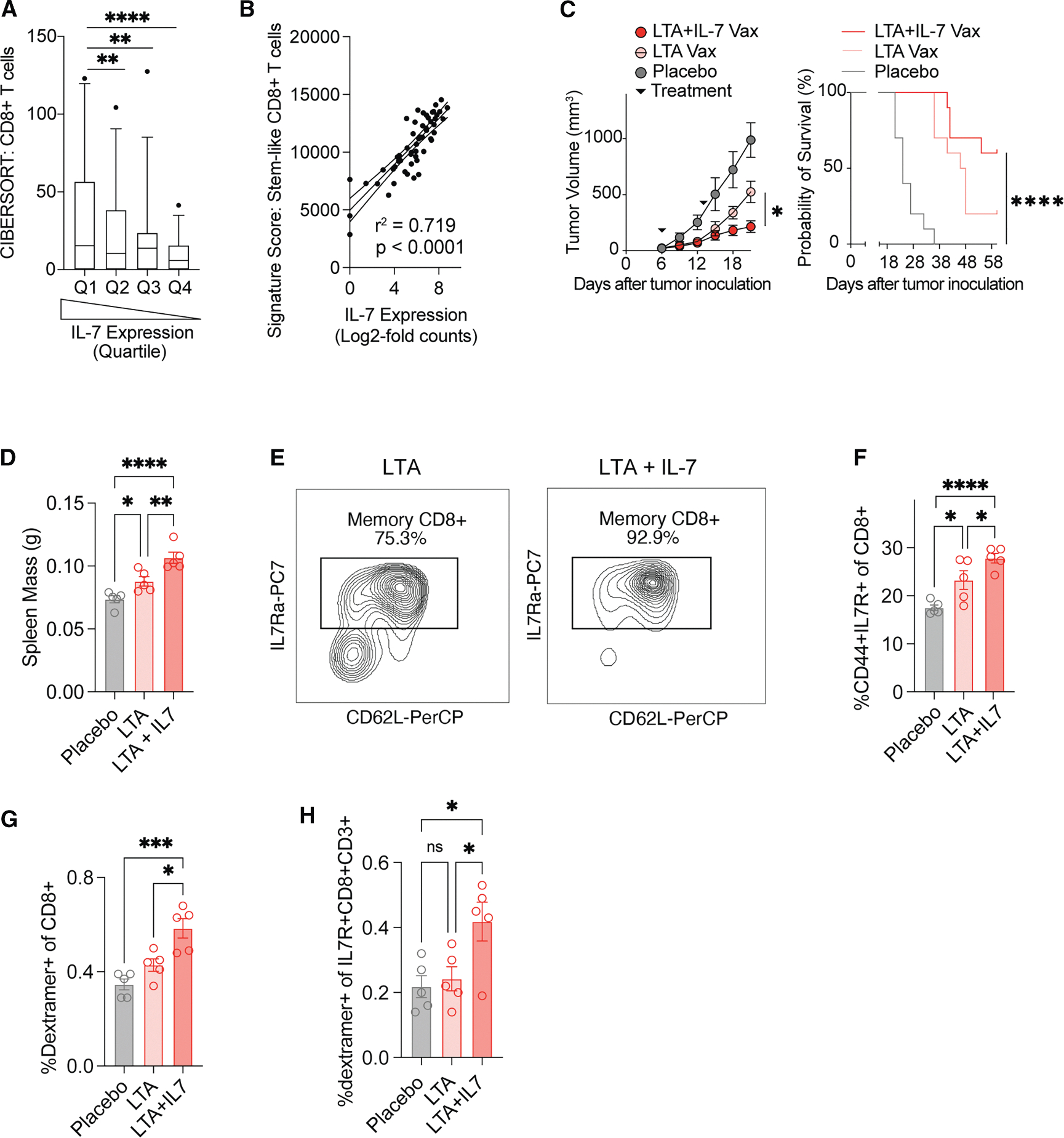
IL-7+antigen co-encoded mRNA vaccines improve T cell expansion and vaccine efficacy (A) Proportion of CD8^+^ T cells by quartile of IL-7 gene expression imputed from bulk RNA-seq measurement of 55 patient samples from^37^ using CIBERSORTx. (B) Linear correlation between IL-7 expression and a gene expression signature of stem-like CD8^+^ T cells from.^38^ Gene signature scores were assigned using ssGSEA. (C) Mice bearing B16-LTA tumors were treated on d6 and 13 with 5 μg of LTA (pink), co-encoded LTA+IL-7 (red) or placebo LNP (gray). Tumor growth curves are depicted (left) along with matched survival data (right). Growth curve statistical comparison by Student’s t test, and survival by Mantel-Cox test. (D) Spleen masses on d25 following vaccination as in (A). (E) Representative flow cytometry plots depicting the CD8^+^ T cell memory compartment from the spleens of two mice treated with either LTA or LTA+IL-7 vaccines on d15 following tumor challenge. (F) Proportion of CD8^+^ T cells with an effector memory phenotype (CD44^+^ IL-7R^+^) in the spleens of mice treated with placebo LNP (gray), LTA vaccine (pink), or LTA +IL-7 vaccine (red). (G) Enrichment of LTA immunodominant epitope-dextramer-positive CD8^+^ T cells on d15 from the spleens of mice following placebo, LTA vaccine, and LTA+IL-7 vaccine treatment. (H) Proportion of dextramer-positive T cells within the memory CD8^+^ T cell population in (G). Data in boxplots are compared using Student’s t test. Data represented in growth curves and bar plots are mean ± SEM; statistical comparison for all bar plots is by ANOVA. *p value < 0.05 and ≥ 0.01, **p value < 0.01 and ≥ 0.001, ***p value < 0.001 and ≥ 0.0001, and ****p value < 0.0001.

The frequency of stem-like and memory phenotype T cells capable of proliferative expansion in tumors is a critical determinant of immunotherapy efficacy across tumor types, including MCC.^31,32^ Moreover, the aging population most at risk for MCC has an increased dependence on T cell memory compared with younger patients, with the infiltration of central memory T cells identified as one of the strongest predictors of immunotherapy response in this population.^31,33,34^ Given the key role that IL-7 signaling plays in the expansion of anti-tumor memory T cells, we hypothesized that it may play a key role in sustaining immune responses in MCC patients and represent a target for improving vaccine efficacy in this disease.^35^ To assess this hypothesis, we tested the relationship between *IL7* gene expression and the infiltration of CD8^+^ T cells and T cells with stem-like transcriptional states in a published cohort of bulk RNA-sequencing data from 55 patients with MCC.^36^ Using CIBERSORTx to assess CD8^+^ T cell infiltration, we found that patients in the highest quartile of *IL7* expression had significantly enhanced CD8^+^ T cell infiltration of tumors compared with any other quartile (Figure 4A).^39^ Moreover, applying single-sample Gene Set Enrichment Analysis (ssGSEA), we found a strong relationship between *IL7* expression in tumors and a published signature of stem-like CD8^+^ T cells (Figure 4B).^38,40^ Given these findings, we hypothesized that mRNA vaccines that co-encode the LTA with IL-7 would improve vaccine-specific memory T cell expansion and efficacy compared with LTA-only vaccines. Prior to *in vivo* use, IL-7 protein expression from co-encoded mRNA vaccines was confirmed by ELISA following transfection into HEK 293T cells (Figure S5B). We next treated mice bearing single-cell-cloned LTA-expressing B16 tumors with 10 μg LTA mRNA (antigen only) or co-encoded LTA+IL-7 (antigen + IL-7) or matched LNP-only control on d6, 13 and 20. Compared with both placebo and antigen-only vaccines, co-encoded antigen + IL-7 vaccines improved tumor control and survival endpoints (Figure 4C). To investigate the mechanism of improved efficacy and characterize changes in antigen-specific memory, we repeated vaccination in non-tumor-bearing mice with two doses on d1 and d7, followed by sacrifice and examination of vaccine-draining lymph nodes and spleens on d15. We observed an increase in the mass of spleens in antigen + IL-7 compared with antigen only or placebo vaccines (Figure 4D), as well as increased formation of IL-7Ra-expressing memory and IL-7Ra^+^ CD44^+^ effector memory phenotype T cells (Figure 4F). Further, using a dextramer for an established LTA epitope (IAPNCYGNI; H2-K^b^),^41^ we identified an increase in antigen-specific T cells in antigen+IL-7 spleens compared with both placebo and antigen only vaccines (Figure 4G), suggesting that memory signal co-encoding improves the expansion of antigen-specific T cells. Finally, within the memory CD8^+^ T cell compartment, antigen + IL-7 vaccines significantly improved the expansion of antigen-specific T cells compared with placebo LNP, while antigen-only vaccines did not (Figure 4H).

## DISCUSSION

Given successful early-phase clinical trials, pandemic-boosted manufacturing capabilities, and evidence of tumor-specific CD8^+^ T cell responses, the current generation of neoantigen-specific mRNA vaccines address some of the challenges that have historically limited the development of therapeutic cancer vaccines. We highlight tumor antigen essentiality and the sustainment of memory T cell responses as two remaining barriers and offer a framework for addressing them via mRNA vaccines that target antigens that are essential for tumor cell survival and co-encode T cell memory and survival signals, including IL-7. We tested the immunogenicity of our vaccine *in vivo* and demonstrated mechanistic improvement by the addition of IL-7 in modified B16 tumor models that are otherwise resistant to immunotherapy, including a uniform cure rate with PD-1+vaccine combinations in a model with limited capability for antigen loss. Because tumor-specific features of MCPyV+ MCC may differ from this model and given historical limitations in mouse-human translation of immunotherapies, we focused further validation efforts on demonstrating proof-of-principle efficacy in MCC patient samples, including antigen-specific expansion, activation, and proliferation; IFN-*γ* secretion; and HLA-matched tumor cell killing.

MCC, an understudied and lethal cutaneous malignancy, represents an exemplary use case for this combined strategy. Despite its historical rarity, our data suggest that MCC incidence is rapidly increasing in multiple demographics in the United States, including in younger patients without frank evidence of immunosuppression, although the causes for this increase remains unclear. Moreover, although ICI has improved the care of patients with advanced and metastatic disease, therapies with durable effect beyond this are lacking. Given that clinical immunohistochemical testing for MCPyV is widely practiced, the selection of virus-positive patients for treatment is clinically tractable. Further, because the LTA has high sequence conservation across patients, vaccine design can be “off-the-shelf,” enabling neoadjuvant treatment approaches in which vaccine is given prior to surgical resection, with pathologic response evaluated at the time of surgery and continuation of treatment in the adjuvant phase to eliminate residual disease. Given the high rate of recurrence of MCC in patients with resected disease, vaccination to prevent regrowth of micrometastatic disease may offer a high potential for efficacy and a favorable risk:benefit ratio. Additionally, the evidence that vaccination enhances response to PD-1 ICI suggests the potential to improve current standard-of-care approaches by sensitizing metastatic disease to aPD-1 in either the front-line or treatment-resistant settings. The potential for this vaccine approach to translate into clinical benefit for MCC patients across a variety of treatment settings—and potentially for select high-risk groups for prophylaxis—is thus significant.

The induction of memory and sustained T cell responses is a significant gap in the construction of the current generation of therapeutic cancer vaccines. Co-encoding IL-7 with the MCC LTA antigen is both a targeted strategy for the aging MCC population in whom antigen-specific T cell frequency may limit response as well as a general strategy for improving T cell memory expansion and sustaining vaccine response. Although prior clinical trials of systemic IL-7 administration were disappointing, substantial literature supports its central role in sustaining long-lasting T cell immunity. We hypothesize that the prior lack of clinical efficacy may have been due to out-of-sequence signaling in which proliferation and memory signals were provided in the absence of appropriate innate immune stimulation, a relevant tumor antigen and co-stimulation—a stark contrast to the present design. Moreover, although systemic IL-7 administration was generally well tolerated, we hypothesize that local administration in the appropriate context of innate and antigen stimulation will limit the risk of systemic toxicity. Although we considered the rationale for IL-7-containing vaccines specifically in MCC, our data provide a rationale for replicating this approach in other antigen-containing tumor vaccines to improve T cell expansion and durable immunologic memory. Beyond IL-7, the strategy of signal co-encoding could be adapted to include single or multiple accessory and adjuvant signals for immune cell expansion, memory formation, and functional enhancement, depending upon the vaccine target and known defects in host immunity.

In summary, we present an mRNA vaccine for the prevention and treatment of virus-associated MCC—a rare but lethal cancer with high unmet clinical need. This strategy may benefit patients in the neoadjuvant, adjuvant, and metastatic settings and overcome resistance to PD-1 ICI. Further, it may be used prophylactically in highly selected groups of patients, such as CLL patients, at high risk to develop MCC. The principles that underlie our vaccination approach: cancer-essential antigen targeting and co-encoding of immunostimulatory and T cell memory signals may, furthermore, be applied broadly to create mRNA cancer therapies capable of inducing effective, sustained responses.

### Limitations of the study

Limitations of the current study and areas for future research include the need for validation in a virus-dependent MCC model and testing in additional patient samples. Therapeutic vaccinations were tested predominately in d6 tumors, which, while palpable, were small. This approach is consistent with an initial strategy of clinical development in the hybrid neoadjuvant-adjuvant setting but underscores the need for future testing in tumors at different stages. Notably, only a single immunodominant epitope was tested via tetramers in patients and by dextramers in mice. Future studies may focus on full deconvolution of the epitopes that are stimulated by LTA vaccination, including a range of HLA types, biaxial validation of binding to control for high background in some tetramer stains, and MHC-I blocking experiments to test for HLA-independent killing mechanisms in patient-matched tumor cell killing experiments. Although the expansion of LTA-specific T cells was observed in the patients tested, it remains to be seen how universal this phenomenon is and whether LTA-specific T cells could be expanded from the blood of healthy donors. Moreover, although LTA + IL-7 vaccines improved systemic expansion of LTA-specific CD8^+^ T cells with memory phenotypes, the effects of these vaccines at the site of muscle injection and on the tumor microenvironment need to be more fully characterized.

## RESOURCE AVAILABILITY

### Lead contact

Further information and requests for resources and reagents should be directed to and will be fulfilled by the lead contact, Jeffrey J. Ishizuka (jeffrey.ishizuka@yale.edu).

#### Materials availability

All plasmids and reagents described are available upon reasonable request.

#### Data and code availability

- Single-cell RNA-seq data discussed in this publication have been deposited in NCBI’s Gene Expression Omnibus and are accessible through the GEO Series accession number GSE305197 (https://www.ncbi.nlm.nih.gov/geo/query/acc.cgi?acc=GSE305197).
- Original western blot images have been deposited in Mendeley at https://data.mendeley.com/datasets/cbndpwhw73/1 (DOI: 10.17632) and will be publicly available as of the date of publication.
- Microscopy data reported in this paper will be shared by the lead contact upon request.
- Any additional information required to reanalyze the data reported in this paper is available from the lead contact upon request.

## STAR★METHODS

### EXPERIMENTAL MODEL AND STUDY PARTICIPANT DETAILS

#### Cell lines

HEK 293T cells and B16-F10 cells were authenticated by STR profiling and tested for mycoplasma contamination every six months.

#### *In vivo* animal studies

All animal studies were conducted with Yale Institutional Animal Care and Use Committee approval, protocol #2023–203207. All experimental animals were wildtype female C57BL/6 mice aged 7–12 weeks, with the exception of aged-mice experiments where mice aged 68 weeks were used. All mice were purchased from Jackson Laboratories (Bar Harbor, ME). Mice were housed in groups of up to 5 littermates and were kept under standard conditions with ad lib diet. For all experiments, mice were randomized *a priori* to treatment or control group by cage number. Unless otherwise specified in the detailed STAR Methods section or main text, mice used in all experiments had not undergone any prior intervention of received any prior experimental drug.

#### Studies involving human participants

All studies involving human participants and human tissue samples was performed with approval of the Yale Institutional Review Board (IRB), protocol #0609001869. All participants signed an IRB approved informed consent prior to tissue/blood donation or collection of patient data. There was no randomization of study participants. All available demographic information for human subjects is reported in Table 1, Patient Cohort Characteristics. Patients were not matched by sex or gender given limitations on sample size and availability.

### METHOD DETAILS

#### mRNA development, validation and efficacy evaluation in mice

##### Generation of an LTA-Expressing murine model

There is currently no available, transplantable murine model comparable to human MCC and no analogous cancer that expresses the viral antigens of interest; therefore, B16-F10 mouse melanoma cells were modified to ectopically express a truncated form of the MCPyV LTA to serve as a model. A consensus sequence of the truncated LTA was identified by comparison with previously sequenced human MCC tumors and optimized based on the known protein functional domains. This yielded a DNA template coding for LTA residues 1–258.^40^ Restriction sites NHEI and MLUI were added to flank the sequence, and a traditional Kozak sequence was added prior to the start codon. A version of this sequence with and without an FLAG tag (DYKDDDDK) was generated so that the target protein could be identified by multiple markers. This template sequence that encodes the truncated LTA was then synthesized into a pUC57 plasmid with ampicillin resistance by GenScript (Piscataway, NJ). Resultant plasmids were then digested using restriction enzymes NHEI and MLUI (New England Biolabs, Ipswich MA) to extract the truncated LTA template. Cas9 was then excised from a pLX311 vector plasmid (Addgene, Watertown MA) using the same restriction enzymes and the truncated LTA template was ligated into the pLX311 placing it in front of an EF1a promoter. A subset of pLX311 plasmids with excised cas9 but no coding region inserted in front of the promoter served as an empty vector (EV) control. Plasmids were transformed and expanded in Top10 *E. coli* and recovered using a Qiagen (Hilden, Germany) MiniPrep kit. Plasmids for LTA and EV were tested by transfection into HEK 293T cells using Polyplus (Illkirch, France) JetOptimus and expression was confirmed by western blot using both LTA directed antibodies (Santa Cruz Biotechnology, Dallas Tx) and FLAG tag antibodies (Sigma, St. Louis MO). Following confirmation of expression, Lentivirus containing the LTA and EV plasmids was produced in HEK 293T cells using a psPAX2 vector as previously described. B16-F10 mouse melanoma cells were then transduced with LTA or EV lentivirus followed by blasticidin selection. Stable expression of the LTA was confirmed by western blot in two of the transduced lines, designated LTA1 and LTAF2, and lack of expression was confirmed in the EV control line. In certain experiments, a single cloned version of the LTA expressing cell line LTAF2 was isolated using limiting dilution and used for *in vivo* experiments. This model is designated LTASC2.

##### Development of a functional LTA encoding mRNA

Using the consensus sequence for the truncated LTA referenced above as a template, additional necessary components of a functional mRNA were added. A T7 promoter sequence was added immediately following the first restriction site to facilitate *in vitro* transcription. For the 5′ untranslated region (UTR), a synthetic sequence was chosen based on high performance in a translation efficiency screen performed by Cao et al.^21^ A signal peptide sequence from the SARS-CoV-2 spike protein was added following the start codon to enhance translation efficiency and assist with trafficking.^22^ The 3′ UTR sequence from the human hemoglobin subunit alpha 1 gene was used due to its short length and success in previous mRNA functional constructs.^23^ Finally, a 100 base polyadenylation sequence was added following the 3′ UTR. This sequence was then commercially synthesized into a pUC57-Simple plasmid by GenScript. The template DNA portion of the plasmid was excised by digestion with restriction enzymes MLUI and XBAI (NEB) and separated from the base plasmid by gel electrophoresis. The template DNA was then purified from the gel using a Zymoclean Gel DNA recovery kit (Zymo Research, Irvine CA). This purified, double stranded, linearized template DNA was then used for mRNA production by *in vitro* transcription using the HiScribe T7 High Yield RNA kit with addition of Trilink (San Diego, CA) CleanCap modified guanosine capping and N1-MethylPseudo-UTP modified uracil base for increased stability and translational efficiency of the final product. Following *in vitro* transcription, the mRNA was purified using a Monarch RNA cleanup kit (NEB) and was stored at −80° until use. The functionality of this mRNA product was tested *in vitro* by transfection into HEK 293T cells using lipofectamine 2000 (Thermo, Waltham MA). Western blot was used to confirm expression of the truncated MCPyV LTA (Figure S3). The mRNA was encapsulated into lipid nanoparticles (LNP) for *in vivo* delivery. LNPs containing SM-102, 1,2-DSPC, cholesterol, and DMG-PEG (Cayman Chemical, Ann Arbor MI) in a lipid molar ratio of 50:10:38.5:1.5 were assembled and incorporated with mRNA using a rapid solvent injection mixing technique. To accomplish this, mRNA was diluted in a 50mM sodium acetate solution at pH 5.0, and was then stirred in a sterile container at 650rpm and the LNP mixture was rapidly injected using a 29 gauge needle into the acidic solution and mixed for 30 min. Placebo injections were created using all the same components, but mRNA was not added. The resulting LNP solution was then dialyzed against 100 volumes of sterile PBS three separate times at 4° using a Slide-A-Lyzer 3.5K MWCO dialysis cassette (Thermo). Purified LNPs were then drawn into sterile 1mL syringes and stored at 4° until use. In certain experiments, an additional IL-7 mRNA was produced by the same method described above using the human IL-7 DNA template sequence. Confirmation of IL-7 mRNA functionality was performed by ELISA (R&D). This mRNA was co-encapsulated in LNPs with LTA mRNA as an adjuvant and used for *in vivo* experiments.

##### Mouse experiments

Seven to twelve-week-old female C57BL/6J mice (Jackson Laboratories, Bar Harbor ME) were used for all *in vivo* experiments, with the exception of aged mice experiments, for which mice were aged to 68 weeks prior to use. For all tumor experiments, syngeneic modified B16-F10 murine melanoma cells were implanted by subcutaneous injection of one million cells in 200 μL of sterile HBSS on the right flank. Tumor volume was calculated using the modified ellipsoid formula V_T_ = (L*W^2^)/2 where L represents tumor length and W represents tumor width. Measurements were taken every three days starting on day 6 or day 9 following tumor engraftment unless otherwise stated. All tumors were confirmed palable prior to mRNA treatments. Humane endpoints resulting in euthanasia included tumor volume greater than 2000 mm^3^, ulceration greater than 25% of the tumor surface area, bleeding ulceration, lethargy, or dehydration. mRNA vaccine treatment was delivered by intramuscular injection into the left gastrocnemius muscle at variable dose and schedule, but constant concentration of 0.15μg/μL. For combination therapy experiments, 200 μg of anti-PD1 antibody (Clone 29F.1A12, Bioxcell, Lebanon NH) or isotype control (Clone 2A3) in 200 μL of sterile PBS was injected intraperitoneally on day 6, 9, and 12. All experiments were performed under IACUC-approved protocol, 2023–203207.

For *ex vivo* analysis of mouse tumors and lymph nodes, euthanasia was performed with isoflurane overdose 15 days following tumor engraftment and tumors and/or lymph nodes were dissected. Tumor draining lymph nodes from the right inguinal region and vaccine draining lymph nodes from the left inguinal region were routinely collected. Tissues were digested using collagenase IV and DNase I followed by mechanical dissociation over a 70 μm cell strainer (Corning, Corning NY). Once a single cell suspension was obtained, cells were stained for viability and with fluorescently labeled surface protein antibodies for detection by flow cytometry. Intracellular staining was also achieved using the eBioscience intracellular fixation and permeabilization buffer set (Thermo). Flow cytometry was performed on a CytoFLEX S machine (Beckman Coulter, Brea CA), and FlowJO V10.9 software was used to analyze data. In a separate experiment, antigen specific CD8^+^ T cells were quantified by flow cytometry using LTA specific dextramers (H2Kb IAPNCYGNI, Immudex, Copenhagen, Denmark). Western blot analysis for *ex vivo* LTA expression was also performed by processing the tumor single cell suspension. Dead cells were excluded by magnetic bead selection using a Miltenyi (Bergisch Gladbach, Germany) Dead Cell Removal kit. CD45^+^ cells were similarly excluded using magnetic bead selection (Stemcell). Live tumor cells were then lysed and antibodies for MCPyV LTA and B-actin were used for protein detection.

#### Evaluation of mRNA efficacy in patient samples

##### *Human* ex vivo *vaccination*

Following informed consent under IRB approved protocol #0609001869, peripheral blood and tumor tissue was collected from MCPyV positive MCC patients to assess the potential efficacy of the LTA vaccine. Tumor tissue was taken from the operating room at time of resection, and processed into single cell suspension under sterile conditions by mechanical dissociation over a 70 μm cell strainer. Primary tumor cells and WAGA (HLA-A2+ immortal MCC cell line) cells were maintained in RPMI (Thermo) medium with 10% serum and 1% pen/strep. Prior to use in killing assays, MCC tumor cells were stimulated with 2ng/mL IFNγ (Stemcell, Vancouver Canada) for 24 h. Peripheral blood mononuclear cells (PBMC) were isolated by density gradient centrifugation using Lymphoprep (Stemcell) medium. Isolated PBMCs were then sorted into CD14^+^ and CD14^−^ populations using magnetic bead selection coordinated to CD14 antibodies (Stemcell). The CD14^+^ population was then divided into one million cell aliquots and frozen for future use or maintained in RPMI medium with 10% serum and 1% pen/strep. In order to polarize the monocytes into monocyte derived dendritic cells (moDC), media was supplemented with 120ng/mL GM-CSF (Stemcell) and 70ng/mL IL-4 (Stemcell) for six days. On day 6, moDCs were transfected with LTA mRNA or placebo using lipofectamine 2000 and were supplemented with 10ng/mL TNF-a (Stemcell) and 1 μg/mL PGE_2_ (Stemcell). One day after transfection, the CD14^−^ population of PBMCs was reintroduced to the co-culture in a 10:1 ratio, and the culture was supplemented with 20ng/mL IL-7 (Stemcell) and 20u/mL IL-2 (Stemcell). The cycle was repeated every 7 days, and new antigen or placebo loaded moDCs were added to the co-culture. Three days following each moDC pulse, the media was collected for determination of IFNγ release by ELISA (R&D, Minneapolis MN). Every 7 days, 10% of the co-culture was used to assess cell populations and activation markers by flow cytometry using the same protocol as described above. On day 14 and 21, killing assays against patient matched MCC tumor cells or HLA-A2 positive WAGA MCC cells in HLA-A2 positive patients were performed. Prior to killing assays, MCC tumor cells were labeled with CellTrace Violet (Thermo) according to manufacturer protocol. Tumor cells and PBMCs from LTA mRNA or placebo co-cultures were plated together in u-bottom 96 well plates at a 5:1 effector-to-target ratio for 24 h. The media was harvested to determine IFNγ release by ELISA, and the cells were counter-stained with viability dye and cell death in CellTrace+ cells was assessed by flow cytometry. In HLA-A2 positive patients, LTA specific tetramers (SMFDEVDEA, NIH Tetramer Core) were used to detect antigen specific CD8^+^ T cell populations in co-culture on day 0, 7, and 10. For tetramer staining, 500,000 cells were taken from LTA mRNA and placebo co-cultures and were pre-treated with 50nM dasatinib to stabilize TCRs. Cells were then stained with tetramers and surface markers for CD3 and CD8 and evaluated by flow cytometry.

##### Cell enrichment and 10× sample preparation for single cell RNA sequencing

Cultured cells were collected stained with TotalSeq anti-human hashtags C0251- C0258 (Biolegend), viability dye (zombie red, Biolegend 423109) and anti-human CD45-FITC (clone HI30, Biolegend 304038) and enriched for live cells and up to 50% immune cells using BD FACS Aria II. Sorted cells were then resuspended to 700–1200 cells per ul and barcoded for multiplexed single cell sequencing using 10× Genomics 5′ v2 chemistry (10× Genomics, PN-1000263).

### QUANTIFICATION AND STATISTICAL ANALYSIS

#### Processing of patient genetic and transcriptomic sequencing data

##### Single cell RNA sequencing and 10× sample alignment

Single cell RNA sequencing libraries were sequenced on Illumina NovaSeq at read length of 150bp using paired end sequencing and depth of 300 million reads per sample. Cell Ranger software v6.0.1 was used to align the 5′ single cell sequencing reads in FASTQ files to reference GRCh38–2020-A, count gene expression, capture antibody hashtags and assemble V(D)J transcript sequences.

##### Single cell RNA and TCR sequencing analysis

Seurat R package was used to perform single cell antibody hashtag demultiplexing, quality control, clustering and visualizations. Specifically, the HTODemux function from Seurat package was used to assign each cell with its corresponding hashtag labeling (s) and based on the assignment, the cell is classified to be either a singlet, doublet or negative. Singlets are kept for further analyses, and they were assigned to their condition of origin. Quality control was done based on nCount_RNA (the total number of molecules detected within a cell), nFeature_RNA (the number of unique genes detected within a cell), percent_mt (percentage of genes traced back to the mitochondrial genome). Normalization, scaling, PCA and unsupervised clustering were also performed using Seurat and cell type annotation for each cluster was performed based on the DGE results by FindAllMarkers. UMAP was performed and used as the primary visualization projection. Clonotype analysis with TCRseq data was performed using scRepertoire. Specifically, each T cell was assigned with its corresponding clonotype when possible. T-cells with the same clonotype were grouped together to create a clonotype specific gene expression matrix which was visualized using bar plot and heatmap. For comparison of LTA vaccine-expanded with virus-positive and virus-negative clonotypes from other patients, CDR3b sequencings from a published cohort of Merkel cell carcinoma samples^42^ were analyzed using GLIPH2.^30^ Briefly, the 24 CDR3b sequences enriched by LTA vaccine were merged with the two groups (virus positive and negative) separately and were input into the GLIPH2 online portal (http://50.255.35.37:8080) with the parameters: GLIPH2 algorithm, reference version 2.0, reference CD8, all_aa_interchangeable as YES. An LTA vaccineenriched clonotype was considered a public clonotype if it shared a cluster with any clonotypes from either the virus positive of negative groups. The match strength (final_score) for the 14 public clonotypes were compared between virus positive and virus negative samples.

##### Analysis of published MCC RNAseq cohort

CIBERSORTx was applied to a published dataset of MCC patient samples^36^ using the LM22 signature set and the online portal (https://cibersortx.stanford.edu). ssGSEA (module v10.0.01) was applied to the same dataset using the signatures noted in the manuscript using the online platform GenePattern. IL-7 expression was grouped by quartile and compared with inferred immune infiltration and signatures using linear regression in GraphPad Prism version 10.

#### Statistical analysis

Unless otherwise specified, a *p*-value of 0.05 was used as a cutoff for statistical significance. In all cases, asterisks were used to denote the following: * = *p*-value <0.05 and ≥0.01, ** = *p*-value <0.01 and ≥0.001, *** = *p*-value <.001 and ≥0.0001, **** = *p*-value <.0001. Graphpad Prism 10 was used to plot data and perform statistical tests including Student’s t test, ANOVA, and Mantel-Cox test for survival as indicated in figure legends. Student’s t tests were unpaired, two-sided unless otherwise described. ImageJ software was used to perform western blot quantification.

## Supplementary Material

1

SUPPLEMENTAL INFORMATION

Supplemental information can be found online at https://doi.org/10.1016/j.celrep.2025.116359.

## Figures and Tables

**Table 1.** Patient cohort characteristics

Identifier	Age	Stage	Concurrent Hematologic Malignancy	Prior Treatment

1	68	pIIIB	N	surgery, radiation
2	73	pI	N	surgery, radiation
3	76	pIIIB	Y	aPD-1 ICI
4	72	recurrent pIV	N	surgery, radiation, aPD-1 ICI

De-identified description of the features of patients included in this study.

**KEY RESOURCES TABLE:** 

REAGENT or RESOURCE	SOURCE	IDENTIFIER

Antibodies		

MCPyV Large T Antigen (CM2B4)	Santa Cruz Biotechnology	Cat#sc-136172
M2 anti-FLAG (DYKDDDDK)	Sigma	Cat#F3165
Anti-PD1 (29F.1A12)	Bioxcell	Cat#BE0273
Rat IgG2a Isotype (2A3)	Bioxcell	Cat#BE0089
LTA Specific Tetramer (SMFDEVDEA)	NIH Tetramer Core	N/A
TotalSeq anti-human hashtag (C0251-C0258)	Biolegend	Cat#394661
Mouse LTA Specific Dextramer (IAPNCYGNI	Immudex	Custom Dextramer

Bacterial and virus strains		

Top10 *E. Coli*	Thermo	Cat#C404003

Biological samples		

Patient Derived Tumor Cells	This Paper	N/A
Patient Derived Peripheral Blood Monocytes	This Paper	N/A

Chemicals, peptides, and recombinant proteins		

Restriction Enzyme NHEI	New England Biolabs	Cat#R3131
Restriction Enzyme MLUI	New England Biolabs	Cat#R3198
JetOptimus	Polyplus	Cat#55-250
Restriction Enzyme XBAI	New England Biolabs	Cat#R0145
Lipofectamine 2000	Thermo	Cat#11668019
SM-102 LNP Kit	Cayman Chemical	Cat#702620
CleanCap AG	Trilink	Cat#W-7113
N1-MethylPseudo-UTP	Trilink	Cat#W-1081
Human IFNy	Stemcell	Cat#78020
Lymphoprep	Stemcell	Cat#18061
Human GM-CSF	Stemcell	Cat#78190
Human IL-4	Stemcell	Cat#78045
Human TNF-a	Stemcell	Cat#78068
Human PGE2	Stemcell	Cat#72192
Human IL-7	Stemcell	Cat#78053.1
Human IL-2	Stemcell	Cat#78036
CellTrace Violet	Thermo	Cat#c34557

Critical commercial assays		

MiniPrep	Qiagen	Cat#27104
Zymoclean Gel DNA recovery kit	Zymo Research	Cat#D4001
HiScribe T7 High Yield RNA Kit	New England Biolabs	Cat#E2040
Monarch RNA Cleanup Kit	New England Biolabs	Cat#T2050
Human IL-7 ELISA	Thermo	Cat#EHIL7
Intracellular fixation and permeabilization kit	Thermo	Cat#88-8824-00
Dead Cell Removal Kit	Miltenyi	Cat#130-090-101
CD45^+^ Magnetic Bead Selection Kit	Stemcell	Cat#18945
CD14^+^ Magnetic Bead Selection Kit	Stemcell	Cat#17858
Human IFNγ ELISA	R&D	Cat#DIF50C

Deposited data		

Original Western Blot Images	Mendeley	https://data.mendeley.com/datasets/cbndpwhw73/1
Raw and Processed Single Cell RNAseq, CITEseq, TCRseq Data	This paper	GEO: GSE305197

Experimental models: Cell lines		

B16-F10 Murine Melanoma	ATCC	ID# CRL-6475
HEK 293T	ATCC	ID# CRL-3216
WAGA (HLA-A2+ immortal MCC cell line)	James DeCaprio	N/A

Experimental models: Organisms/strains		

Mouse: C57BL/6J	Jackson Laboratories	RRID:IMSR_JAX:000664
Oligonucleotides		
LTA Consensus Sequence Residue 1–258	Laude et al.^19^	N/A
High Performance 5′ UTR Sequence	Cao et al.^21^	N/A
SARS-CoV-2 Spike Protein Signal Peptide	Zhang et al.^22^	N/A
Human Hemoglobin Subunit Alpha 1 3′ UTR	Wollner et al.^23^	N/A

Recombinant DNA		

Customized pUC57-LTA Plasmids	GenScript	Cat#SD1176
pLX311-Cas9 Plasmid	Addgene	Cat#118018
psPAX2 Plasmid	Addgene	Cat#12260

Software and algorithms		

FlowJO V10.9	BD Biosciences	www.flojo.com
Prism 10	Graphpad	www.graphpad.com
CIBERSORTx	Newman et al.^39^	N/A
R 4.2.0	R Core Team (2022)	https://www.R-project.org/
R studio	Posit team (2025)	http://www.posit.co/
Seurat V5	Satija Lab	https://doi.org/10.1038/s41587-023-01767-y
GLIPH2	Huang et al.^30^	http://50.255.35.37:8080

Other		

Slide-A-Lyzer 3.5K MWCO Dialysis Cassette	Thermo	Cat#66330
70μm Cell Strainer	Corning	Cat#431751

## Highlights

- mRNA vaccines against an essential Merkel cell viral oncoprotein enhance immunity
- Vaccination is effective in mouse models and Merkel cell carcinoma patient samples
- Antigen loss limits efficacy in mouse models but not in MCC patients
- Co-encoded IL-7 mRNA boosts antigen-specific T cell memory and vaccine efficacy
