# Supplementary material for: Targeting an essential viral oncoprotein with an IL-7-enhanced mRNA vaccine induces durable immunity to Merkel cell carcinoma
Source: Cell Rep. Author manuscript; Available in PMC 2025 Nov 25. (PMC12646823; doi:10.1016/j.celrep.2025.116359)
Supplement: 1 [file NIHMS2120133-supplement-1.pdf]

Cell Reports, Volume *44*

Supplemental information

Targeting an essential viral oncoprotein

with an IL-7-enhanced mRNA vaccine

induces durable immunity to Merkel cell carcinoma

Alexander Frey, Kathryn Clulo, Yuewei Fei, Therese Cordero Dumit, Frankie Scallo, Jerry William Allen, Emily Chang, Curtis J. Perry, Lena V. Wirth, Daniel Jacobs, David A. Braun, Marcus W. Bosenberg, Thuy T. Tran, James Clune, Harriet M. Kluger, Kelly Olino, and Jeffrey J. Ishizuka

SUPPLEMENTAL INFORMATION

Figures S1–S5

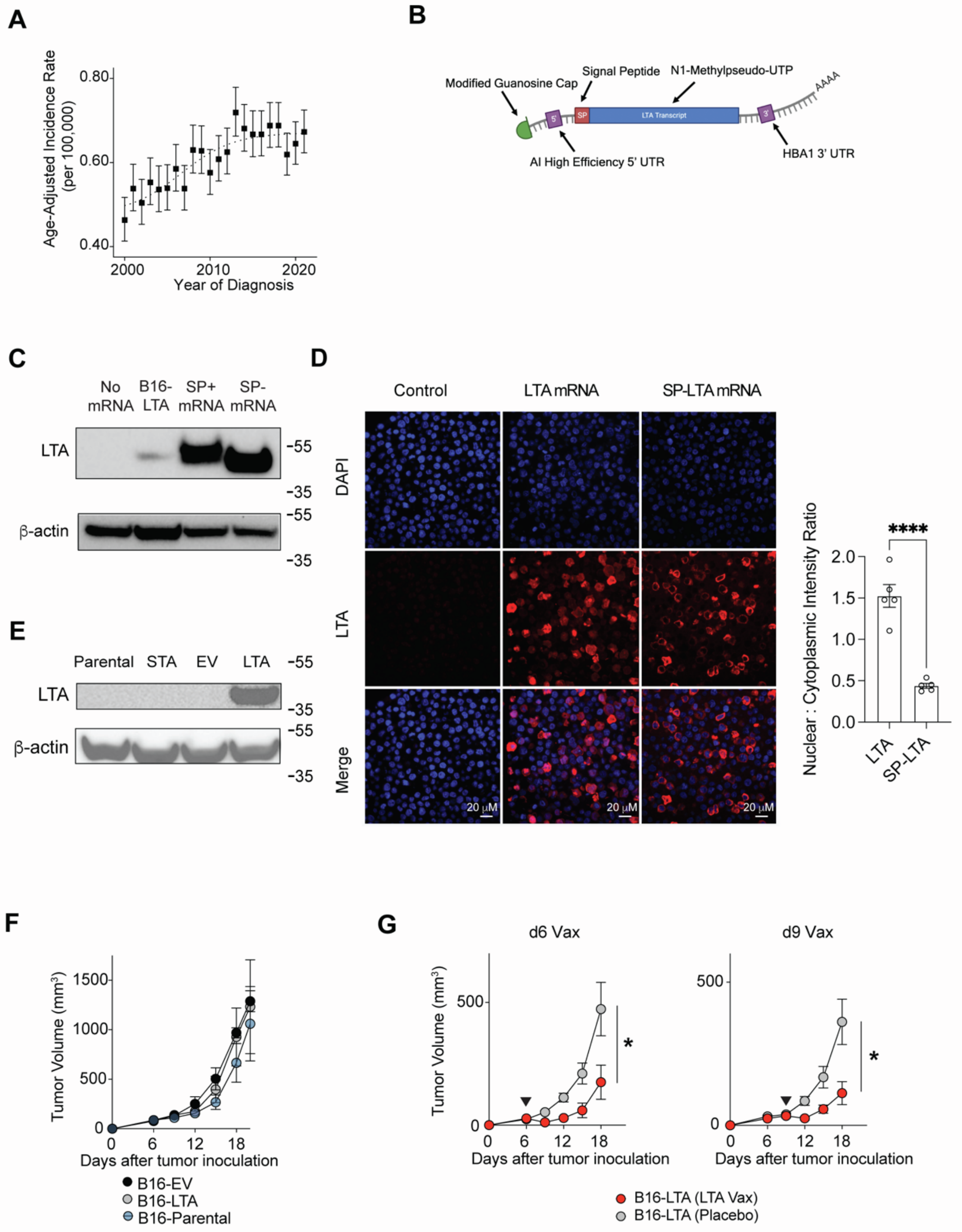
A
Age-Adjusted Incidence Rate (per 100,000)
0.80
0.60
0.40
2000
2010
2020
Year of Diagnosis
B
Modified Guanosine Cap
Signal Peptide
N1-Methylpseudo-UTP
AAAA
SP
LTA Transcript
AI High Efficiency 5' UTR
HBA1 3' UTR
C
No mRNA
B16-LTA
SP+ mRNA
SP- mRNA
LTA
β-actin
-55
-35
D
Control
LTA mRNA
SP-LTA mRNA
DAPI
LTA
Merge
20 µM
Nuclear : Cytoplasmic Intensity Ratio
****
2.0
1.5
1.0
0.5
0.0
LTA
SP-LTA
E
Parental
STA
EV
LTA
LTA
β-actin
-55
-35
F
Tumor Volume (mm³)
1500
1000
500
0
Days after tumor inoculation
B16-EV
B16-LTA
B16-Parental
G
d6 Vax
d9 Vax
Tumor Volume (mm³)
500
0
6
12
18
Days after tumor inoculation
*
B16-LTA (LTA Vax)
B16-LTA (Placebo)

Figure S1. Development of an LTA-targeting mRNA vaccine and murine model for testing.
(A) Incidence rate for Merkel cell carcinoma through 2021 from SEER data. Dots represent fitted points with 10% confidence intervals. Dashed lines represent fitted curve.
(B) Schematic design of LTA-targeting mRNA vaccine.
(C) Western blot of LTA expression in a test cell line (HEK293T) following transfection with and without signal peptide inclusion.
(D) Immunofluorescence of LTA expression within nuclear and cytoplasmic fraction of HEK293T cells (left) with accompanying quantification (right) following transfection of 2 ug of either LTA or SP-LTA mRNA.
(E) WB depicting LTA expression in Parental, Small T Antigen-expressing (STA), Empty Vector control (EV) and LTA-expressing (LTA) B16 tumor cells.
(F) Tumor growth of B16 tumor cells modified to express LTA (B16-LTA, gray), empty vector control (EV, black) compared with parental cells (blue). For tumor growth and survival experiments, 1 million tumor cells were injected subcutaneously.
(G) Effects of a single dose of 15 μg vaccine on d6 and on d9 after tumor implantation.
Data represented in growth curves and bar plots are mean ± SEM, all statistical comparisons are by Student's t test. * = p-value <0.05 and ≥0.01, ** = p-value <0.01 and ≥0.001, *** = p-value <.001 and ≥0.0001, **** = p-value <.0001.

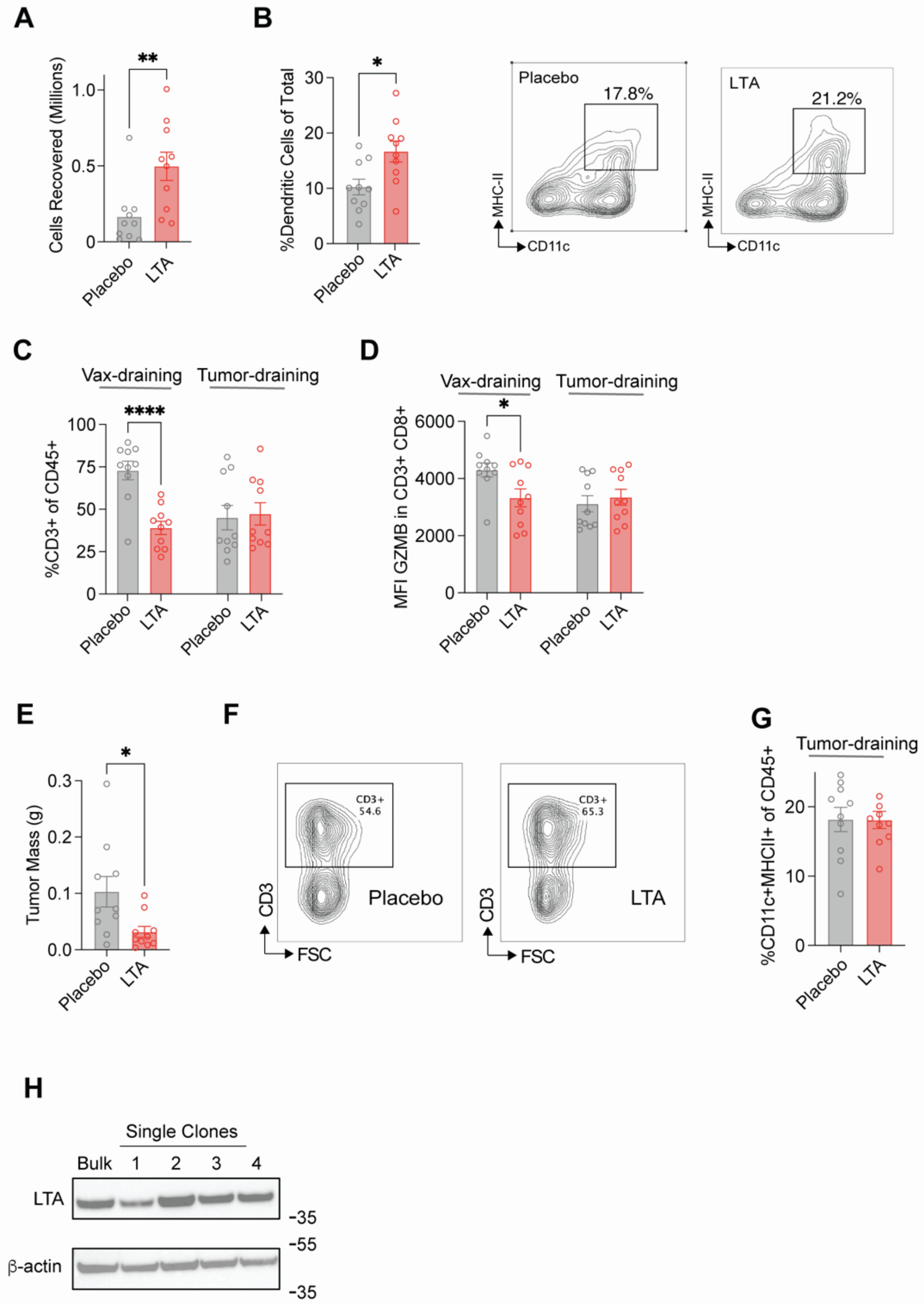
A
B
C
D
E
F
G
H
Cells Recovered (Millions)
%Dendritic Cells of Total
Placebo
LTA
17.8%
21.2%
MHC-II
CD11c
Vax-draining
Tumor-draining
%CD3+ of CD45+
MFI GZMB in CD3+ CD8+
Tumor Mass (g)
CD3+
54.6
65.3
CD3
FSC
%CD11c+MHCII+ of CD45+
Single Clones
Bulk
LTA
β-actin

Fig. S2. Supporting flow cytometry and single-cloned murine model generation data.
Mice were treated and lymph node and tumor tissues were processed for flow cytometry as in **Fig 2** with data from placebo- and vaccine-treated mice depicted in gray and red, respectively.
(A) Expansion of immune cells in vaccine- compared with placebo-draining lymph nodes. Total cells recovered from vaccination site-draining lymph node dissection and analyzable by flow cytometry are depicted in millions of cells.
(B) Percentage of $CD45^+$ cells identified as dendritic cells and gating strategy in vaccine-draining lymph nodes from placebo and LTA vaccine-treated mice.
(C) $CD3^+$ cells as a percentage of $CD45^+$ immune cells in vaccine-draining and tumor-draining lymph nodes.
(D) MFI of GZMB in $CD3^+CD8^+$ T cells.
(E) Mass of tumors extracted for flow cytometry from placebo- and vaccine-treated mice.
(F) Gating strategy for $CD3^+$ T cells from tumors.
(G) Dendritic cells in tumor-draining lymph nodes as a percentage of $CD45^+$ live cells.
(H) LTA expression in single cell clones from LTA-expressing B16-LTA bulk population measured by western blot.
Data represented in bar plots are mean ± SEM, all statistical comparisons are by unpaired, two-tailed test. * = p-value <0.05 and ≥0.01, ** = p-value <0.01 and ≥0.001, *** = p-value <.001 and ≥0.0001, **** = p-value <.0001.

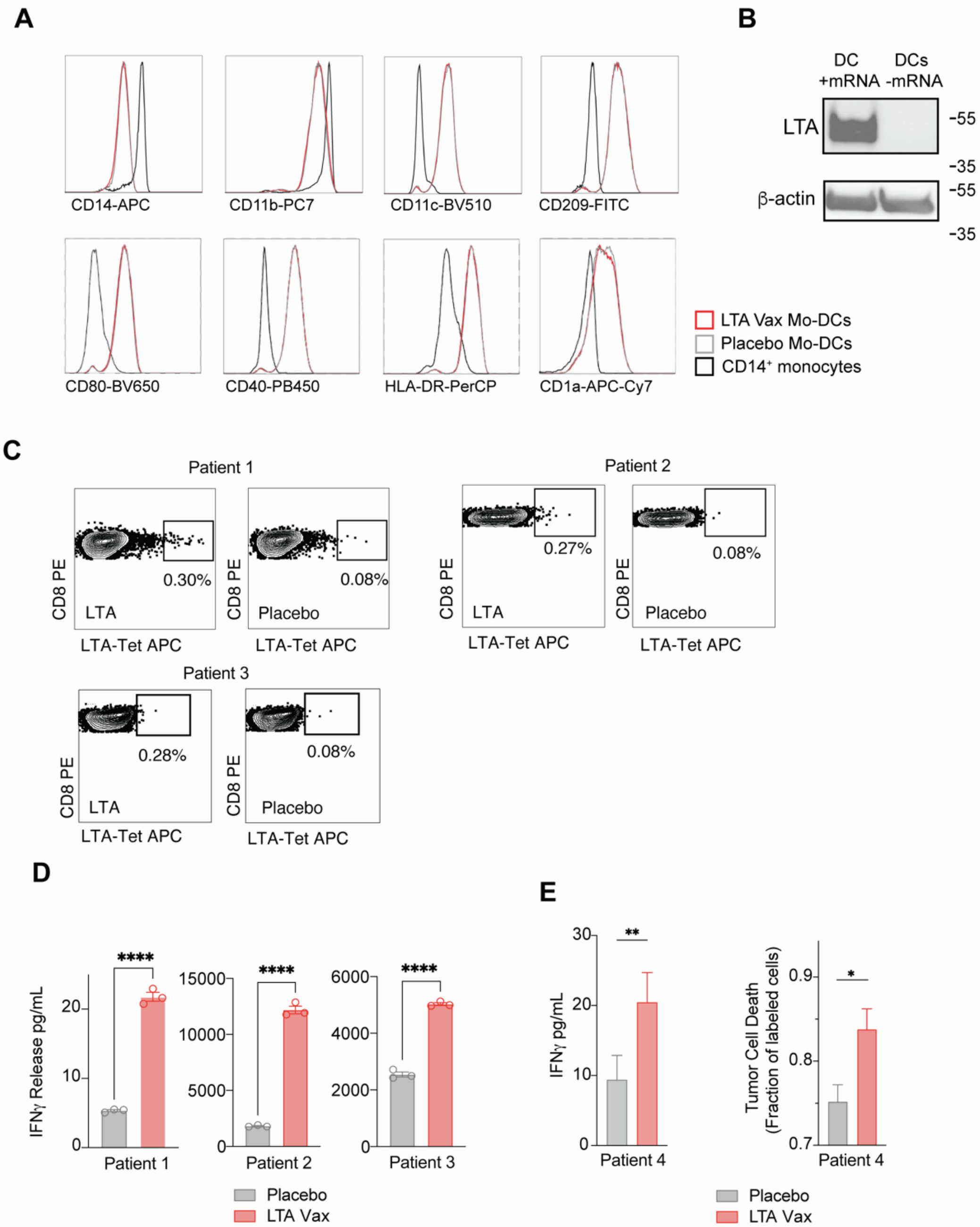
A
CD14-APC
CD11b-PC7
CD11c-BV510
CD209-FITC
CD80-BV650
CD40-PB450
HLA-DR-PerCP
CD1a-APC-Cy7
LTA Vax Mo-DCs
Placebo Mo-DCs
CD14⁺ monocytes
B
DC +mRNA
DCs -mRNA
LTA
β-actin
-55
-35
-55
-35
C
Patient 1
Patient 2
Patient 3
CD8 PE
LTA-Tet APC
LTA
Placebo
0.30%
0.08%
0.27%
0.08%
0.28%
0.08%
D
IFNγ Release pg/mL
Patient 1
Patient 2
Patient 3
****
Placebo
LTA Vax
E
IFNγ pg/mL
Tumor Cell Death (Fraction of labeled cells)
Patient 4
**
*
Placebo
LTA Vax

Fig. S3. Supporting data for patient *ex vivo* vaccination experiments.

(A) Histograms of flow cytometry of monocyte (CD14), myeloid (CD11b), dendritic cell (CD11c, CD209, CD1a) and dendritic cell activation (CD80, CD40, HLA-DR) markers in $CD45^{+}$ live cells from $CD14^{+}$ enriched monocyte-derived dendritic cell cultures activated using TNF and PGE_2.

(B) Expression of LTA protein following mRNA vaccine transfection in moDCs as measured by western blot.

(C) Representative tetramer staining for MCPyV+ MCC Patients 1, 2 and 3.

(D) ELISA results for detection of IFNγ following 24-72 hours co-culture of LTA vaccine-expanded patient T cells stimulated with LTA-transfected moDCs (red) or placebo-expanded T cells stimulated with placebo-transfected moDCs (gray).

(E) (Left) ELISA for detection of IFNγ following 24 hours of co-culture of LTA vaccine-expanded or placebo T cells cultured with patient-matched primary tumor cells. (Right) Killing of patient-matched primary tumor cells by LTA-expanded (red) or placebo (gray) patient T cells.

Data represented in bar plots are mean ± SEM, all statistical comparisons are by Student's t test. * = p-value <0.05 and ≥0.01, ** = p-value <0.01 and ≥0.001, *** = p-value <.001 and ≥0.0001, **** = p-value <.0001.

A

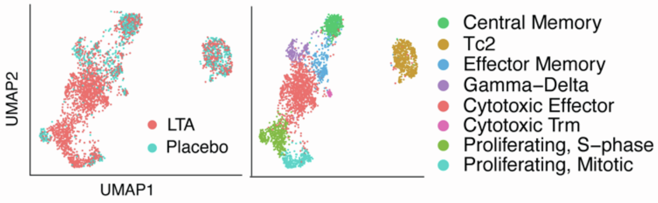

B

Population	Key Differentially Expressed Transcripts
Central Memory	*SELL, IL7R, TCF7, LEF1, CXCR4, CCR7, CD44*
Tc2	*GATA3, IL5, IL13, IL9R, CCR4*
Effector Memory	*GZMK, GZMH, PRF1, IL7R, CD161, KLF2, XCL1*
Gamma-Delta	*TRDV1, TRDC, TRGV3, 4, 5, 8, TRGC1*
Cytotoxic Effector	*GZMA, PRF1, NKG7, IFNG, CCL5, IL7R-low*
Cytotoxic Trm	*GZMB, PRF1, NCR1, XCL1, ZNF683, ITGAE, CXCR6*
Proliferating, S-Phase	*MCM2, 3, 4, 5, 6, 7, PCNA, TYMS, EF21*
Proliferating, Mitotic	*MKI67, CCNB1, 2, CDK1, AURKA, B, PCNA*

C

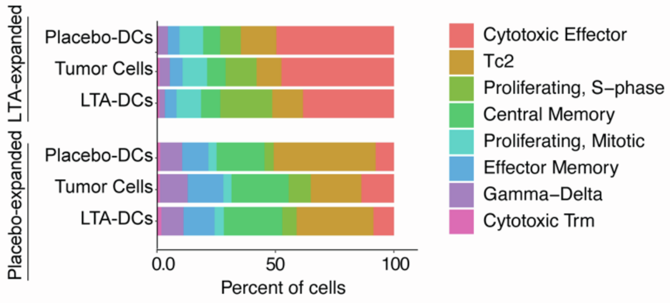

D

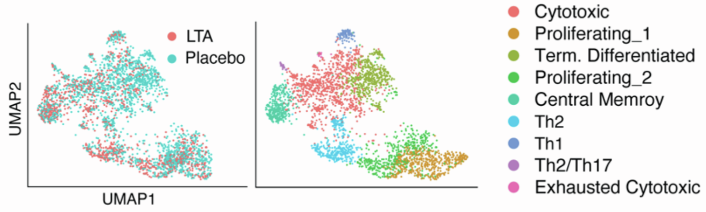

E

Population	Key Differentially Expressed Genes
Cytotoxic	*GZMA, GZMB, GZMM, CCL5, CCL17, CCL21*
Proliferating_1	*MKI67, PCNA, CDK1, 2,4, CCNA2, MCM2, 3, 5, 6, 7*
Terminally Differentiated	*LAG3, HAVCR2, CTLA4, CD2, CD7, CCL2,3,5*
Proliferating_2	*MCM2, 3, 5, 6, 7, LDHA, LDHB, PCNA*
Central Memory	*IL7R, CCR7, SELL, TCF7, LEF1, CD44*
Th2	*IL13, GATA3, IL4R, IL9R, CCR4, TCF7,*
Th1	*IFNG, TBX21, CCL4, CCL5, JUN, RUNX2, TNF*
Th2/Th17	*IL13, IL17RB, IL9R, IL26, STAT3, TNF, IL32*
Exhausted Cytotoxic	*GNLY, GZMB, PRF1, LAG3, TIGIT, HAVCR2, PDCD1*

F

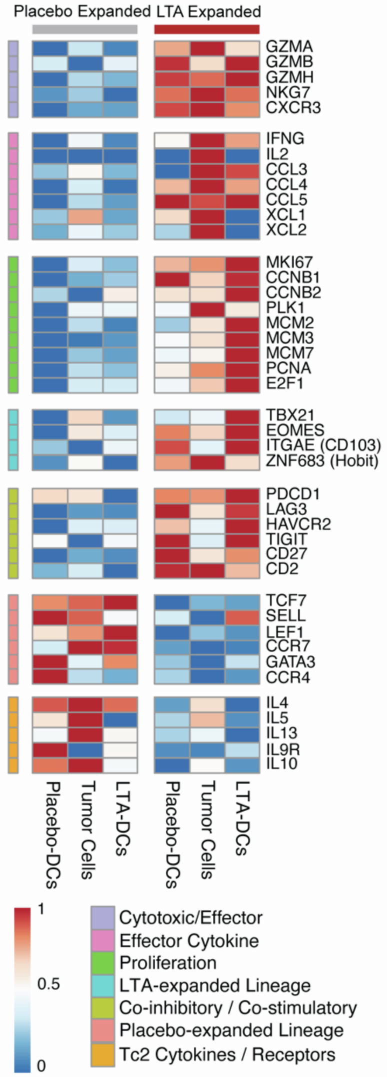

Fig. S4. Supporting data for patient single cell sequencing experiments.
(A) UMAP plots of $CD8^+$ T cells expanded by LTA vaccine or placebo LNPs (left). Cluster identities corresponding to enriched populations (center and right).
(B) Cluster markers corresponding to (**A**).
(C) Proportions of each $CD8^+$ T cells in each cluster.
(D) UMAP plots of $CD4^+$ T cells expanded by LTA vaccine or placebo LNPs (left). Cluster identities corresponding to enriched populations (center and right).
(E) Cluster markers corresponding to **(D)**.
(F) Transcriptional programs enriched following LTA vaccination or placebo LNP treatment and stimulation with Placebo-DCs, matched primary tumor cells or LTA-DCs.

A

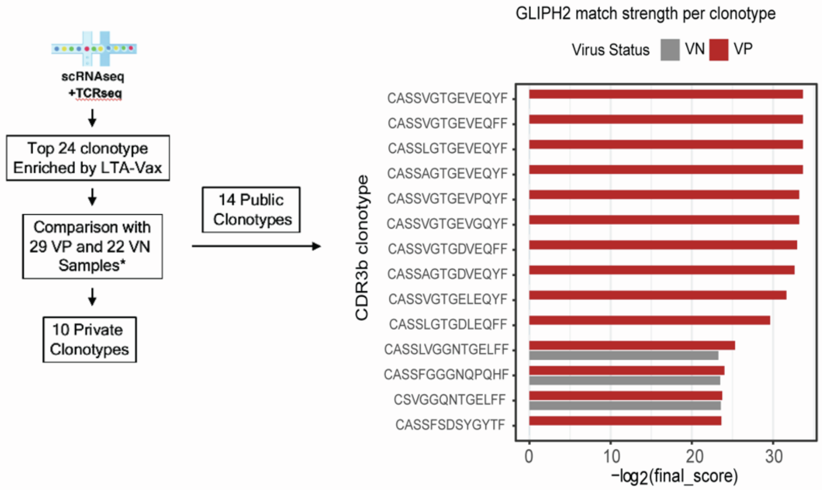
scRNAseq
+TCRseq
Top 24 clonotype Enriched by LTA-Vax
Comparison with 29 VP and 22 VN Samples*
10 Private Clonotypes
14 Public Clonotypes
GLIPH2 match strength per clonotype
Virus Status VN VP
CDR3b clonotype
CASSVGTGEVEQYF
CASSVGTGEVEQFF
CASSLGTGEVEQYF
CASSAGTGEVEQYF
CASSVGTGEVPQYF
CASSVGTGEVGQYF
CASSVGTGDVEQFF
CASSAGTGDVEQYF
CASSVGTGELEQYF
CASSLGTGDLEQFF
CASSLVGGNTGELFF
CASSFGGGNQPQHF
CSVGGQNTGELFF
CASSFSDSYGYTF
0
10
20
30
−log2(final_score)

B

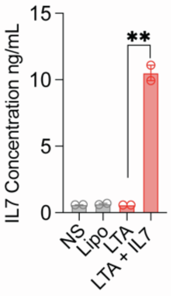
IL7 Concentration ng/mL
15
10
5
0
**
NS
Lipo
LTA
LTA + IL7

Fig. S5. Supporting data for IL-7 + LTA vaccines.

(A) Workflow for comparing vaccine-expanded T cell receptor (TCR) clonotypes with clonotypes from patients with virus positive (VP) and virus negative (VN) MCC tumors (Left). Of 24 expanded clonotypes, 14 public clonotypes were identified. Vaccine-expanded clonotypes preferentially overlapped with published TCRs from virus-positive compared with virus-negative patients using overlap via GLIPH2 (Right).

(B) ELISA of IL-7 expression in HEK293T cells following transfection with lipofectamine alone, LTA and LTA + IL-7 mRNA.

Data represented in bar plots are mean ± SEM. Statistical comparison in (B) is by Student's t test. * = p-value <0.05 and ≥ 0.01, ** = p-value <0.01 and ≥ 0.001, *** = p-value $<.001$ and ≥ 0.0001, **** = p-value $<.0001$.
